# Evaluation of 17 years of MERIN (Meningitis and Encephalitis register in Lower Saxony, Germany) surveillance system: participants acceptability survey, completeness and timeliness of data

**DOI:** 10.1186/s12913-023-10482-y

**Published:** 2024-01-11

**Authors:** Anna Łuczyńska, Konrad Beyrer, Ina Holle, Armin Baillot, Masyar Monazahian, Johannes Dreesman, Elke Mertens, Sophie Rettenbacher-Riefler

**Affiliations:** 1 European Centre for Disease Control and Prevention (ECDC), ECDC Fellowship Programme, Field Epidemiology Path (EPIET), Stockholm, Sweden; 2 Department for Microbiology, Infection Protection, Hospital Hygiene and Epidemiology of Infectious Diseases, Public Health Agency of Lower Saxony, Hanover, Lower Saxony Germany

**Keywords:** Surveillance, Polio-eradication, Evaluation, Enterovirus, Meningitis

## Abstract

**Background:**

A Meningitis and Encephalitis Surveillance (MERIN) was implemented in 2003 in Lower Saxony, Germany as an alternative to acute flaccid paralyses surveillance, as the latter did not reach WHO sensitivity criteria. The system provides information on circulating enterovirus (EV) serotypes by focussing on patients with suspected aseptic meningitis, encephalitis or acute flaccid paralysis and contributes to the national surveillance in documenting polio free status. MERIN is based on voluntary participation of hospitals. Therefore, our evaluation focusses on acceptability of the system’s objectives and performance, and identifying areas for improvement.

**Methods:**

To assess acceptability, 32 contributing hospitals were invited to an online-based survey (11/2021 to 01/2022) to rate the MERIN objectives, laboratory’s performance, their workload, modes of processes and communication. Ideas for improvement were collected in open fields. In addition, data completeness and timeliness of laboratory diagnostics were assessed.

**Results:**

Of 32 hospitals, 21 responded (66% response rate), sending 30 questionnaires, 25 from pediatric and 5 from neurological departments. High levels of satisfaction with the communication (≥ 96%), timeliness (≥ 81%), and distribution of the results (≥ 85%) were reported, 97% of participants judged the required workload as adequate. The median proportion of eligible patients included in MERIN was 75%. Participants gave rapid and reliable diagnostic testing the highest priority (96%), while monitoring of Germany’s polio-free status was rated the lowest (61%). Providing medical reports digitally as well as regular updates about circulating EV serotypes were identified as areas for improvement. Data completeness of selected variables ranged from 78.3 to 99.9%. Median time between sample collection and arrival at laboratory was 2 days [IQR 1–3], EV diagnostics via PCR took one day [IQR 0–6] and EV isolation on cell culture 11 days [IQR 10–13].

**Conclusion:**

MERIN is a highly accepted surveillance system. Its quality was enhanced further by addressing the suggested improvements such as regular reports on circulating EV serotypes and facilitating digital access to laboratory results.

Our results emphasise the importance of recognizing and considering participants’ motivations and expectations, and addressing their priorities, even if this is not the surveillance system’s main focus.

This approach can be applied to surveillance systems of other non-mandatory notifiable diseases.

**Supplementary Information:**

The online version contains supplementary material available at 10.1186/s12913-023-10482-y.

## Background

Monitoring cases of acute flaccid paralysis (AFP) among children under 15 years of age is considered gold standard for polio surveillance. It is used in many countries to demonstrate the presence or absence of poliovirus circulation and is one element in the certification process of a country towards polio-free status [1, 2]. However, countries that have been polio free for many years or even decades are challenged to maintain the surveillance’s sensitivity criteria of detecting at least one non-polio AFP case per 100 000 children under the age of 15 years per year, required by the Polio Eradication Initiative [1, 3, 4].

The national AFP surveillance in Germany was established in 1998 as a way of demonstrating polio-free status. However, the results of the surveillance proved to be insufficient according to AFP sensitivity criteria [5]. In Germany only 50 to 60% of the expected AFP cases were reported from the clinics (approx. 60 to 70 of 116 AFP cases) [6]. Therefore, an alternative approach was necessary to increase acceptability of the surveillance among clinics and to fulfil polio surveillance requirements.

In 2003 Lower Saxony was the first federal state in Germany to implement an alternative to AFP surveillance system focussing on patients with suspected aseptic meningitis, encephalitis or acute flaccid paralysis – the Meningitis and Encephalitis Register in Lower Saxony (MERIN) hosted by the Public Health Agency of Lower Saxony (NLGA) [6, 7]. In 2010 MERIN became the model for the federal enterovirus surveillance system, which replaced AFP surveillance to demonstrate polio free status of Germany. Hospitals from the federal state of Bremen started joining MERIN in 2011. MERIN offers laboratory diagnostic testing for hospitalized patients with suspected aseptic meningitis or encephalitis. Participating hospitals, mostly paediatric, neurological and internal medicine clinics in Lower Saxony and Bremen, send specimens such as viral swabs, serum samples and cerebrospinal fluid to the virological laboratory of NLGA and receive laboratory diagnostics free-of charge. The medical reports are delivered by post mail or fax. Routine testing is performed for enteroviruses (EV), including polioviruses, but also for e.g. herpes simplex, varicella-zoster, measles, mumps and tick-borne encephalitis viruses via direct pathogen detection (PCR and cell culture) as well as serological analyses. This broad diagnostic coverage was chosen to enhance engagement of clinicians and their participation in the MERIN and thereby increase its acceptability. During the last 10 years (2013–2022), on average 1800 samples from over 700 patients were sent annually by 39 different hospitals. The majority (79%) of the patients were younger than 15 years. More than a half (56%) of meningitis and encephalitis cases were caused by EV. Polioviruses were not detected [8]. Aside from being a stand-alone surveillance system in two federal states, MERIN is also the major contributor to the national German enterovirus surveillance [5].

Evaluation of surveillance systems is essential to improve their performance and should be conducted on a regular basis. The complexity and the different settings in which they are implemented requires surveillance systems to be targeted and context relevant [9, 10]. Since MERIN is based on voluntary contributions of the participating hospitals, our evaluation focusses on acceptability among participants.

This evaluation aims to better understand the surveillance system and its challenges, views and perceptions of the hospitals currently involved in MERIN, with the outlook to identify areas for improvement and formulate and apply specific recommendations.

## Methods

### Data collection via questionnaire

Hospitals were considered active participants if they sent at least one sample during the two years prior to the survey (2019–2020). An online-based survey among actively participating hospitals in MERIN was conducted between November 2021 and January 2022. Participants were invited to complete a web-based questionnaire created with LamaPoll® software (Lamano GmbH, Berlin Germany). The link to the questionnaire was sent via E-Mail. A reminder was sent four weeks after the initial invitation. Electronic informed consent was obtained prior to the start of the survey. Participation was voluntary, anonymous for respondents, and they could withdraw from the study at any time. The completion of the survey required about 15 min.

The aim of the survey was to assess acceptability of the system’s main objectives and performance. For the latter, the questions addressed three major domains: (i) the relations between participating hospital and NLGA laboratory, (ii) the timeliness and modes of processes and (iii) participants’ satisfaction with the MERIN and the amount of their involvement (adapted and modified from participatory AccEPT method adapted to evaluation of surveillance systems [11–13]. Table 1 presents the questionnaire outline in detail.

**Table 1:** Evaluated acceptability elements with associated questions

**Acceptability’s elements**	**Associated questions**	**Relevant question/section in the questionnair**e
**Objective**	Are participants satisfied with the objectives of the surveillance system? Do they consider the objectives important/relevant?	Assessment of the relevance of MERIN objectives
**Performance**		
** Relationship between hospital and NLGA laboratory**	i) Are MERIN participants satisfied with their relation with the NLGA?	• Quality of communication with the NLGA laboratory
** Timeliness and modes of processes**	ii) Are MERIN participants of satisfied with the processes of the MERIN surveillance system?	• Processing time for direct and serological pathogen detection
		• Assessment of the data collection and medical report forms
		• Dissemination of results
		• Satisfaction with the range of diagnostic tests provided
** Participants involvement/satisfaction**	iii) Are MERIN participants satisfied with the surveillance system and their duty?	• Adequacy of workload
		• Proportion of eligible patients submitted to the MERIN

We also collected data on age group, occupation and length of professional experience from the medical personnel who answered the questionnaire on behalf of the participating hospital (respondents).

Questions required binary and/or Likert–scale answers. Each section also provided free-text fields for remarks and ideas of improvement.

### Completeness and timeliness of data

To complement the survey results, data completeness and timeliness were assessed. We restricted the data set to the period 2003–2019, thereby excluding the possible impact of the COVID-19 pandemic. Completeness of variables in the request form (age, gender, postal code, reported symptoms) as well as reporting rates of follow-up forms (a form requesting the treating physician to report final patient outcome) and timeliness between sample collection and arrival at laboratory and from sample arrival to obtaining laboratory test results (EV diagnostic via PCR and via cell culture) was assessed.

### Data analyses

Answers of the Likert scale questions were dichotomized into negative (Likert categories 1–3) and positive (4–6) answers and visualized by graphs showing gradients of red and blue colors, respectively.

Completeness of variables was measured as percentage of completed data fields in reports from 2003 to 2019. Median time between sample arrival in the laboratory and obtaining results for various laboratory tests was calculated.

In order to investigate potential relationships between survey variables and data completeness and identify the most influential variables we created a correlation heatmap for the Spearman rank correlation coefficient and corresponding p-values for all variables paired with each other. Means of the answers were calculated if more than one questionnaire was obtained from the respective hospital. Analyses were carried out in R 4.2.2.

## Results

Seven hospitals were excluded as they had sent less than one sample during the last two years prior to the survey, resulting in 32 actively participating hospitals (29 from Lower Saxony and 3 from Bremen). We received questionnaires from 21 of the 32 hospitals invited to participate. This corresponds to a response rate of 66%. All 21 hospitals were from Lower Saxony. Six hospitals contributed more than one questionnaire. Of these six, one hospital contributed four questionnaires (three from the pediatric ward and one from the neurological ward), one hospital contributed three questionnaires from the same ward and the other four hospitals contributed two questionnaires each from the same ward. A total of 30 questionnaires were used for the final evaluation, of which 25 came from pediatric and 5 from neurological departments, 27 (93%) were answered by chief or senior physicians (Table 2).

**Table 2:** Demographics of the survey respondents, Nov 2021-Jan 2022

	**n**	**%**
**Age (years)**		
18–35	2	7.7
36–59	19	73.1
≥60	5	19.2
Missing	4	
**Occupation**^**a**^		
Chief medical officer	11	37.9
Senior physician	16	55.2
Other clinician	2	6.9
**Years of professional experience**		
2 to < 5	2	6.9
5 to < 10	10	34.5
≥ 10	17	58.6
Missing	1	
**Department**		
Pediatry	25	83
Neurology	5	17

n: number of participants in each category

^a^German translations: Chief medical officer: Chefarzt, Chefärztin, Senior physician: Oberarzt, Oberärztin

**Fig. 1:**
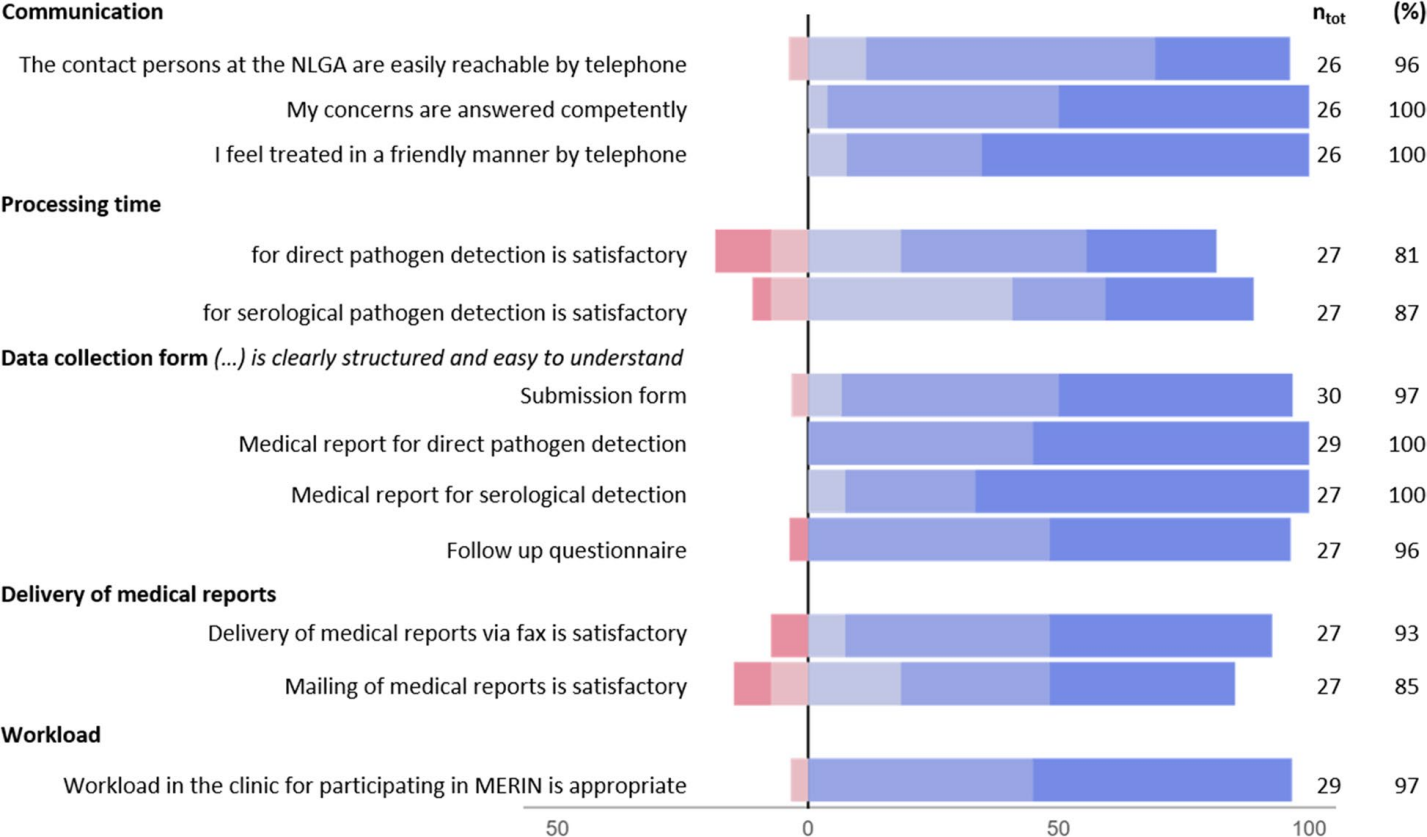
Satisfaction with communication, timeliness of sample processing, workload, data collection forms and the way of delivery of medical reports rated by survey participants, Nov 2021-Jan 2022. ntot: Total number of respondents to the question. (%): proportion of respondents that responded positively to the questions (blue categories, 4–6). The Likert scales used: (1) strongly disagree – (6) strongly agree. Further details on the answers’ distribution are given in Additional file 1

Figure 1 summarizes the results of participants’ satisfaction with communication, timeliness of sample processing, workload, data collection forms and delivery of medical reports. Most of the respondents rated communication with the NLGA positively and were very satisfied with reachability (96%, 25/26) and competence (100%, 26/26) but also with friendliness (100%, 26/26) of the NLGA personnel.

The processing time for direct pathogen detection and for serological pathogen detection was found satisfactory by 81% (22/27) and 89% (24/27) of participants respectively.

The content and formal design of the submission form and medical reports were perceived as clearly structured and easy to understand. The delivery of medical reports by fax was satisfactory for 86% (25/27) of the participants, mailing of medical reports per post for almost 80% of them (23/27). A common issue mentioned by several respondents in the free-text fields was digitalisation. Specifically, the need for electronic transmission of the results was emphasized. Participants also indicated they would appreciate receiving regular reports on circulating EV serotypes.

The workload required for participating in MERIN was rated as appropriate or absolutely appropriate by 97% (28/29) of the participants.

Regarding the correlations, workload perceived as appropriate correlated significantly with satisfaction regarding all investigated aspects of communication: reachability (*ρ* = 0.67, *p* = 0.001), competence (*ρ* = 0.71, *p* = 0.002) and friendliness (*ρ* = 0.73, *p* = 0.001).

**Fig. 2:**
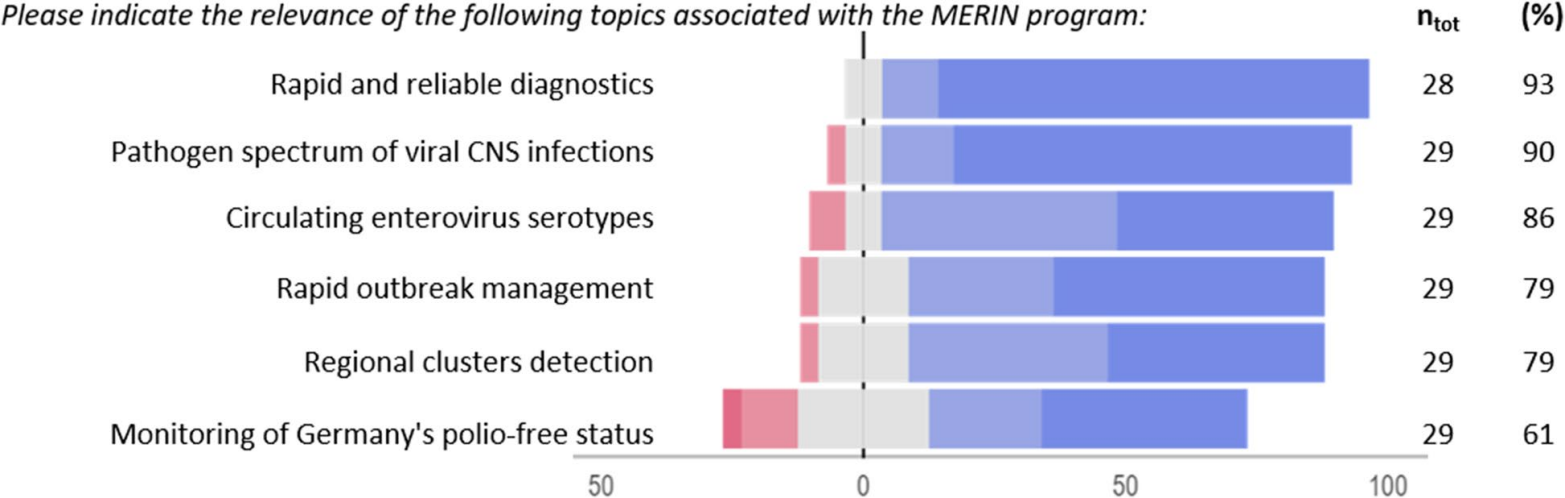
Prioritisation of MERIN objectives from the perspective of the participants, Nov 2021-Jan 2022. ntot: Total number of respondents to the question. (%): proportion of respondents that responded positively to the questions (blue categories, 4–5). The Likert scales used: (1) unimportant, (2) less important, (3) neutral, (4) important, (5) very important

Regarding the relevance of the MERIN objectives, rapid and reliable diagnostic testing was given the highest priority (93%, 26/28), followed by pathogen spectrum of viral CNS infections (90%, 26/29), currently circulating enterovirus serotypes (86%, 25/29), rapid outbreak management (79%, 23/29) and detection of regional clusters (79%, 23/29). Monitoring of polio-free status in Germany was rated the lowest priority, still 61% (17/29) of the participants considered it an important or a very important objective (Fig. 2).

**Fig. 3:**
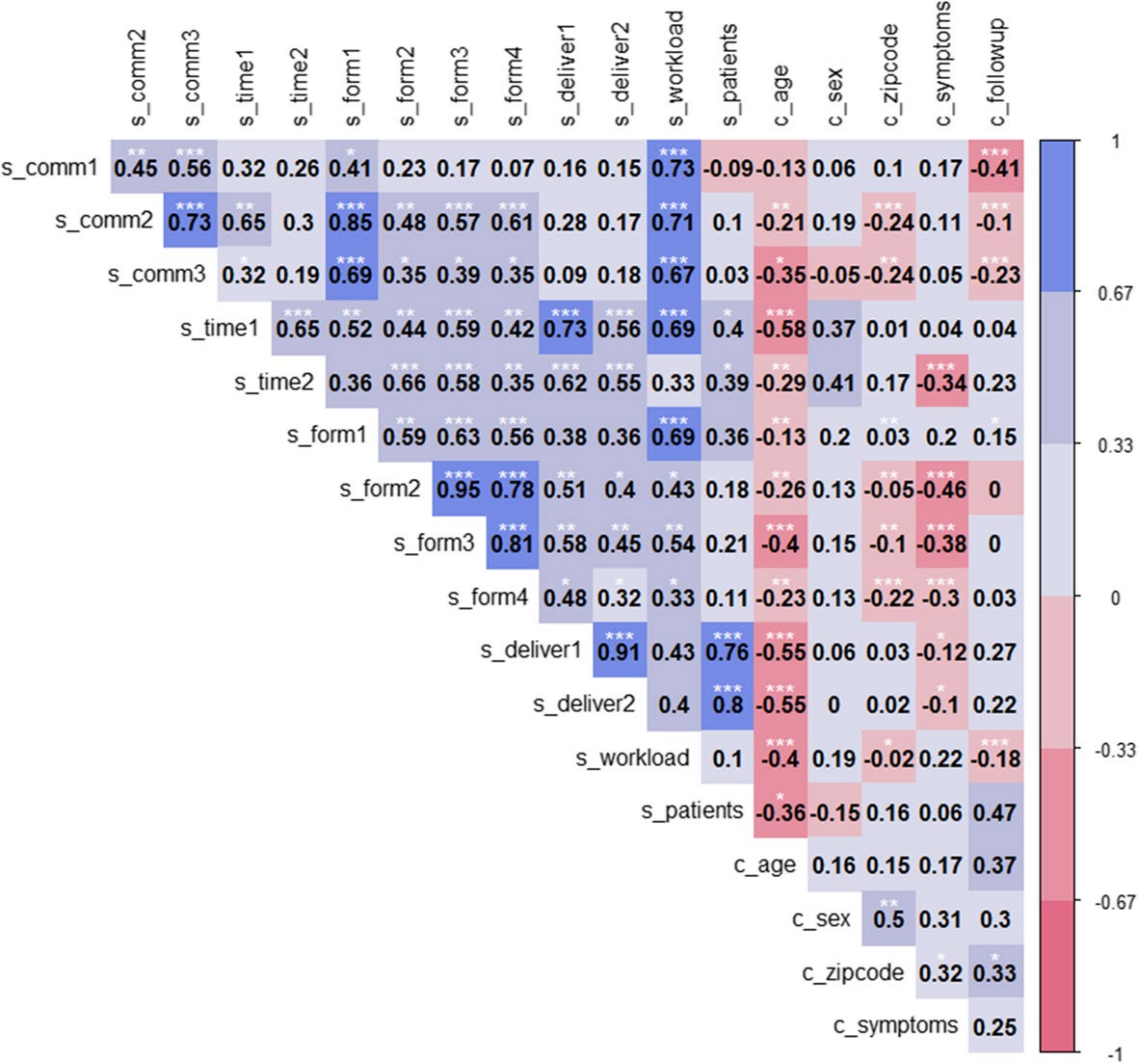
Spearman’s rank correlation heatmap of data completeness (2003–2019) and questionnaire items.Values show the Spearman rank correlation coefficients (rho). Red colours show negative correlations, blue shows positive correlation. Correlations (confidence interval 0.95) with *p* > 0.05 treated as not significant. *P*-values indicated by asterisk: *<0.05, **<0.01, ***<0.001. Survey variables are denoted by “s”, variables used for completeness analyses are denoted by “c”. The variable description is given in the table below

About half (57%; 17/28) of all participants stated that 100% of all their meningitis/encephalitis patients were included in the MERIN. The median proportion of patients included was 75%. This parameter correlated with the level of satisfaction regarding mailing (*ρ* = 0.8, *p* < 0.001) or faxing (*ρ* = 0.76, *p* = 0.001) the medical reports (Fig. 3).

**Table:** **Description of variables used for the correlation heatmap**

Variable	Description/Question in the questionnaire
s_comm1	The contact persons at NLGA are easily reachable by telephone.
s_comm2	My concerns are answered competently.
s_comm3	I feel treated in a friendly manner over the telephone
s_time1	The processing time for direct pathogen detection (e.g. PCR) is satisfactory.
s_time2	The processing time for serological detection is satisfactory.
s_workload	The workload in the clinic for participating in MERIN is appropriate.
s_form1	The submission form is clearly structured and easy to understand.
s_form2	The medical report for direct pathogen detection is clearly structured and understandable.
s_form3	The medical report for serological pathogen detection is clearly structured and easy to understand.
s_form4	The follow-up questionnaire is clearly structured and easy to understand.
s_deliver1	Delivery of medical reports via fax is satisfactory.
s_deliver2	Mailing of medical reports is satisfactory.
s_patients	Percentage of eligible patients that are included in the MERIN.
c_age	Percentage of complete data fields for age (2003–2019)
c_sex	Percentage of complete data fields for sex (2003–2019)
c_zipcode	Percentage of complete data fields for postal code (2003–2019)
c_symptoms	Percentage of complete data fields for symptoms (2003–2019)
c_followup	Reporting rates of follow-up forms (2003–2019)

The reasons most frequently given for not including all suspected cases of aseptic meningitis/encephalitis in the MERIN were either that the laboratory diagnostics was done by another laboratory (64%; 7/11) and “staff forgot/was unaware” (55%, 6/11). The statement “initial clinical picture inconclusive” was selected four times (36%).

The majority (93%, 26/28) of the participants found the offered scope of laboratory diagnostics sufficient. The analysis of two additional viral pathogens for the differential diagnosis of meningitis/encephalitis (“bocaviruses” and “HHV 6”) was suggested in the open field textbox.

Overall, identified potentials for improvement involved online access of medical records and regular reports on currently circulating enterovirus serotypes.

Complementing the survey results, analysis of data completeness of the variables on the submission form showed consistently high values over the years, ranging between 80 and 99,9% across data fields in 2019 (*n* = 748). Reporting rate of follow-up forms was 58% (Additional file 2).

The calculated median time between sample collection and arrival at the laboratory was 2 days [IQR 1–3], for EV diagnostics via PCR 1 day [IQR 0–6] and for EV isolation on cell culture 11 days [IQR 10–13]. Participants who were satisfied with the processing time were also satisfied with delivery of medical reports via fax (*ρ* = 0.73, *p* = 0.003) (Fig. 3).

## Discussion

Efficiency of any surveillance highly depends on stakeholders’ engagement and participation [10]. Therefore, assessing their willingness to participate is crucial, and can contribute to reduced underreporting, improvement of data quality and also identification of clues for system improvement [10, 12]. As highlighted in a systematic review of the current approaches to evaluate surveillance systems [10], the attribute of acceptability should be viewed as an essential factor for the quality of surveillance. It has also been listed by Center for Disease Control and Prevention as one of the main requirements for proper functioning of the system [9].

Our findings demonstrate that MERIN is a successful model for a voluntary-based surveillance. Our investigation revealed a high level of system acceptability as evidenced by participants’ satisfaction with the system’s objectives, their relationship with the laboratory, the timeliness and modes of processes involved as well as their assigned duties related to MERIN. The high participants’ satisfaction was underlined by findings regarding timeliness and completeness of data (age, gender, zip code).

The study showed that a majority of the respondents were highly satisfied with the communication with the NLGA. All investigated aspects of communication (reachability, competency and friendliness) strongly correlated with the MERIN related workload perceived as appropriate. This finding can be seen as an indicator of the good relationship between the participants and the NLGA laboratory, a prerequisite, in our view, for the success of voluntary surveillance of non-mandatory notifiable diseases.

Regarding timeliness and modes of processes, most survey participants indicated that the data collection forms were clearly structured and easy to understand, participants also appreciated the timeliness and wide range of the provided diagnostic testing. Potentials for improvement were identified particularly with regard to electronic transmission of the results and direct and regular access to the surveillance reports. Moreover, satisfaction with medical reports delivery via post mail and fax highly correlated with the proportion of meningitis/encephalitis patients included in MERIN. This finding may suggest that hospitals that are satisfied with the way of delivery are more likely to include all of their eligible patients to MERIN. As a consequence, providing digital medical reports could increase the number of patients submitted.

Regarding surveillance objectives, participating hospitals valued rapid and reliable diagnostics highest, while monitoring polio free status in Germany was rated the lowest. Prompt laboratory diagnosis of an EV infection may reduce antibiotic or antiviral drug usage, limit unnecessary and costly investigations, shorten the length of patient hospitalization and minimize the risk of complications [14–16]. Our findings showed that participants’ needs and expectations relating to system objectives differ from those of the surveillance provider (NLGA). This was demonstrated by an almost reverse pattern of prioritization of the surveillance objectives between the two partners. The acknowledgment of the importance and consideration of the participant’s needs seems to be a critical step towards enhancing the effectiveness of surveillance efforts. Relatedly, understanding the relevance of stakeholder’s perceptions to ensure proper functioning of the surveillance system has been highlighted in previous research [10, 17, 18].

The participants’ input on surveillance system performance does not only foster its improvement, their engagement can also make it more acceptable. The insights obtained from the survey participants contributed to enhancing the MERIN with regular reports on circulating EV serotypes. Likewise, updating the current website was initiated. Participating hospitals were each provided with a written report, summarizing the overall survey results and implemented improvement measures. Lack of electronic reporting was identified as a considerable challenge. In order to retain participants and to expand the system, efforts to simplify access to laboratory results digitally should continue. Over the years, the diagnostic offer has been constantly advanced and adapted in order to accommodate new diagnostic or technological advances. Further improvements will include implementation of molecular typing of EV via next-generation sequencing.

A limitation of our study is that the hospitals that stopped participating in MERIN were not contacted. Identifying the reasons for discontinuing participation and understanding the potential barriers could help identify factors that could enhance hospitals’ involvement in the MERIN. Although a relatively high response rate [19] among the participating hospitals was found in our study, the reasons for non-participation were not further explored. In particular, identifying the reasons for non-participation among the three hospitals in Bremen might be of importance. Lacking representation of this federal state might have introduced non-response bias. In addition, the respondents consisted mainly of experienced medical personnel. This group might not be involved in daily work activities and might have a different view-point than other hospital personnel. Our study could also suffer from social desirability bias since the laboratory diagnostics are offered free of charge. However, it has been shown that social desirability bias is less likely to occur with online surveys than other data collection methods like face-to-face interviews or telephone surveys [20]. Another limitation of our study, common for cross-sectional surveys, is that it offers a snapshot taken at a point in time and does not allow for monitoring of changes. The latter will be addressed by repeated surveys in regular intervals in the future. Elucidating the hospitals’ motivations to leave MERIN retrospectively is challenged by the difficulty of finding staff that was entrusted with the decision at the time. The questionnaire in the current study was specifically designed for active participants, however, more research is needed including follow-up studies of why certain hospitals discontinued MERIN participation as well as non-respondents to the survey to better understand challenges and barriers for participation.

In spite of the listed limitations, our study clearly demonstrates that MERIN is a highly accepted surveillance system. This approach can be transferred to similar projects and can be used for implementation in other settings or to surveillance systems of other non-mandatory reported diseases based on voluntary stakeholder contribution. EV surveillance differs substantially between individual European countries but typically focuses on enteroviruses detected from patients with severe infections presenting with neurological symptoms [21]. Since the notification of EV is non-mandatory in most countries in the EU, including Germany, their surveillance relies on voluntary reporting of clinicians, hospitals or laboratories [5, 21]. Efforts to harmonize and collate surveillance data on the European level have recently been established through the European Non-Poliovirus Enterovirus Network (ENPEN) [21–23].

## Conclusions

MERIN was evaluated as a highly acceptable surveillance system. Identified areas of improvement contributed to enhancing the system with regular reports on circulating EV serotypes and efforts to simplify access to laboratory results. Our results emphasise the importance of recognition and consideration of participant motivations and expectations, provision of high-quality services and regular and effective communication between the partners. With the obtained knowledge, we aim to increase surveillance structure, raise awareness about MERIN among eligible hospitals in Lower Saxony and Bremen and increase participation rate which in consequence could contribute to increase performance of the system. This approach can be easily applied to other settings or to surveillance systems of other non-mandatory notifiable diseases that wish to strengthen the acceptability of their surveillance systems.

### Supplementary Information

**Additional file 1.** Satisfaction with communication, timeliness of sample processing, workload, data collection forms and the way of delivery of medical reports rated by survey participants, Nov 2021-Jan 2022.

**Additional file 2.** Data completeness for each variable (as percentage) on request form and follow up forms over time (2003-2019).

## Data Availability

The datasets used and/or analysed during the current study are available from the corresponding author on reasonable request.

## Abbreviations

AccEPT: Acceptability Participatory Toolkit
AFP: Acute Flaccid Paralysis
ENPEN: European Non-Poliovirus Enterovirus Network
EU: European Union
EV: Enterovirus
HHV 6: Human herpesvirus 6
IQR: Interquartile range
MERIN: Meningitis and Encephalitis Register in Lower Saxony (German translation:Meningitis und Enzephalitis Register in Niedersachsen)
NLGA: Public Health Agency of Lower Saxony (German translation Niedersächsisches Landesgesundheitsamt)
WHO: World Health Organization
