# Supplementary material for: Evaluation of 17 years of MERIN (Meningitis and Encephalitis register in Lower Saxony, Germany) surveillance system: participants acceptability survey, completeness and timeliness of data
Source: BMC Health Serv Res. 2024 Jan 11;24:59. doi: 10.1186/s12913-023-10482-y (PMC10782521; doi:10.1186/s12913-023-10482-y)
Supplement: Supplementary file 1 — Additional file 1. Satisfaction with communication, timeliness of sample processing, workload, data collection forms and the way of delivery of medical reports rated by survey participants, Nov 2021-Jan 2022. [file 12913_2023_10482_MOESM1_ESM.docx]

**Additional File 1.** Satisfaction with communication, timeliness of sample processing, workload, data collection forms and the way of delivery of medical reports rated by survey participants, Nov 2021-Jan 2022

|  | 1 (Strongly disagree) | 2 | 3 | 4 | 5 | 6 (Strongly agree | | Missing |
| --- | --- | --- | --- | --- | --- | --- | --- | --- |
| **Communication** |  |  |  |  |  |  |  | |
| The contact persons at the NLGA are easily reachable by telephone | - | - | 1 (3.8%) | 3 (11%) | 15 (58%) | 7 (27%) | 4 | |
| My concerns are answered competently | - | - | - | 1 (3.8%) | 12 (46%) | 13 (50%) | 4 | |
| I feel treated in a friendly manner by telephone | - | - | - | 2 (7.7%) | 7 (27%) | 17 (65%) | 4 | |
| **Processing time** |  |  |  |  |  |  |  | |
| The processing time for direct pathogen detection (e.g. PCR) is satisfactory. | - | 3 (11%) | 2 (7.4%) | 5 (19%) | 10 (37%) | 7 (26%) | 3 | |
| The processing time for serological detection (e.g. Antibody ELISA) is satisfactory. | 1 (3.7%) | 2 (7.4%) | 2 (7.4%) | 11 (41%) | 5 (19%) | 8 (30%) | 3 | |
| **Workload** |  |  |  |  |  |  |  | |
| The workload in the clinic for participating in MERIN is appropriate. | - | - | - | 1 (3.4%) | 13 (45%) | 15 (52%) | 1 | |
| **Data collection form** *(…)* *is clearly structured and easy to understand* |  |  |  |  |  |  |  | |
| Submission form |  |  | 1 (3.3%) | 2 (6.7%) | 13 (43%) | 14 (47%) | - | |
| Medical report for direct pathogen detection |  |  |  |  | 13 (45%) | 16 (55%) | 1 | |
| Medical report for serological detection |  |  |  | 2 (7.4%) | 7 (26%) | 18 (67%) | 3 | |
| Follow up questionnaire |  |  |  | 1 (3.7%) | 13 (48%) | 13 (48%) | 3 | |
| **Delivery of medical reports** |  |  |  |  |  |  |  | |
| Delivery via fax is satisfactory |  | 2 (7.4%) | - | 2 (7.4%) | 11 (41%) | 12 (44%) | 3 | |
| Delivery via mail is satisfactory |  | 2 (7.4%) | 2 (7.4%) | 5 (19%) | 8 (30%) | 10 (37%) | 3 | |
