# Supplementary material for: Evaluation of 17 years of MERIN (Meningitis and Encephalitis register in Lower Saxony, Germany) surveillance system: participants acceptability survey, completeness and timeliness of data
Source: BMC Health Serv Res. 2024 Jan 11;24:59. doi: 10.1186/s12913-023-10482-y (PMC10782521; doi:10.1186/s12913-023-10482-y)
Supplement: Supplementary file 2 — Additional file 2. Data completeness for each variable (as percentage) on request form and follow up forms over time (2003-2019). [file 12913_2023_10482_MOESM2_ESM.docx]

**Additional File 2.** Data completeness for each variable (as percentage) on request form and follow up forms over time (2003-2019).

|  | 81-100 |
| --- | --- |
|  | 71-80 |
|  | 61-70 |
|  | 51-60 |
|  | 40-50 |
|  | < 40 |
|  | no data |

n: number of data entries in reports from 2003 to 2019

Data presented as percentages (%) except for column n
